# Effects of age on suicide attempts by medication in patients transferred to the emergency rooms of two advanced medical centers: A retrospective chart review of the DJ project

**DOI:** 10.1002/npr2.12367

**Published:** 2023-07-11

**Authors:** Taro Sasaki, Masataka Shinozaki, Aki Nemoto, Yukiko Nagao, Manabu Yasuda, Kazutaka Shimoda, Shiro Suda, Norio Yasui‐Furukori

**Affiliations:** ^1^ Department of Psychiatry Dokkyo Medical University School of Medicine Mibu Japan; ^2^ Department of Psychiatry Jichi Medical University Shimotuke Japan

**Keywords:** age, means, over‐the‐counter drugs, pesticides, suicide attempt

## Abstract

**Aims:**

The means of suicide vary, but in cases of impaired consciousness, it is often difficult to determine the initial treatment because it is not known whether a patient has overdosed or used pesticides or poisons. Therefore, we investigated the clinical characteristics of suicide by medication in patients with suicide attempts who were brought to the emergency department, especially the influence of age.

**Methods:**

Patients with suicide attempts were transported to the two hospitals. There were 96 males (38.4%) and 154 females (61.6%). The mean age was 43.5 ± 20 years, and both males and females were most often in their 20s. Data on sex, age, motive for suicide, means of suicide attempt, psychiatric diagnosis, length of hospital stay, and place of discharge were retrospectively analyzed.

**Results:**

The average age of the patients by means of suicide attempt was 40.5 years for “prescription drugs,” 30.2 years for “over‐the‐counter drugs,” and 63.5 years for “pesticide/poison.” For each means of suicide attempt, there was a significant difference in age among patients with suicide attempts using “prescription drugs,” “over‐the‐counter drugs” and “pesticides/poisons.” There was a statistical bias in the means and reasons for each suicide attempt.

**Conclusion:**

The results showed that the age of patients who used over‐the‐counter medicines and pesticides and poisons varied significantly. It was thought that pesticide use should be considered first, especially when patients aged 50 years and over are brought to the hospital with impaired consciousness due to suicide attempts.

## Abstract 2

Histogram of the age distribution. Gray indicates female, and black indicates male. The top diagram (A) shows the age distribution of suicides committed with prescription drugs, the middle diagram (B) shows the age distribution of suicides committed with over‐the‐counter drugs, and the bottom diagram (C) shows the age distribution of suicides committed with pesticides and poisons. The mean age in patients receiving “prescription drugs” was significantly higher than that in patients receiving “over‐the‐counter drugs,” and the mean age in patients receiving “pesticides/poisons” was significantly higher than that in patients receiving “prescription drugs.” In addition, the mean age of patients receiving “over‐the‐counter drugs” was significantly higher than that of patients receiving “pesticides/poisons.”

## Graphical abstract

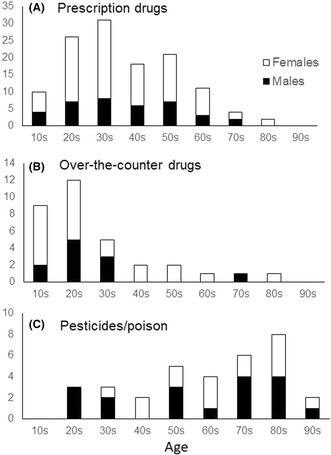

## 1 INTRODUCTION

In 2019, a total of 759 028 (523 883 males and 235 145 females) deaths due to suicide were reported worldwide.^1^ Moreover, rates of suicidal behavior among youth have gradually increased over the past 15 years and continued to rise during the COVID‐19 pandemic. This trend places a burden on mental health services and calls for important developments in the detection of risk and the provision of interventions to reduce risk.^2^ A history of suicide attempts is known to be an important and the most powerful risk factor for suicide.^3^, ^4^ In particular, when a patient with past suicide attempts is brought to the hospital for any kind of suicide attempt, prompt identification of the means of suicide is important to save their life. In psychiatric care, there are many opportunities to encounter patients with psychiatric disorders who have suicidal ideation and self‐harm. Especially in general hospitals with emergency medical services, there are many cases in which patients who have attempted suicide are transported to the hospital and require careful treatment for both physical and mental health issues. In some cases, the details of the patient's circumstances at the time of the suicide attempt are known from family members or acquaintances who were nearby, while in other cases, the patient is found unconscious, and it is unclear what the means of the suicide attempt was. There are various means of suicide, but in the case of impaired consciousness, it is often difficult to determine the initial treatment because it is often not known whether a patient has overdosed (“OD”) or used pesticides/poisonous substances. If the characteristics of the drug can be clarified to some extent based on clinical notes, it will provide important information for initial lifesaving treatment.

Therefore, we investigated the clinical characteristics of suicide attempts by medication in patients brought to the emergency room.

**FIGURE 1:**
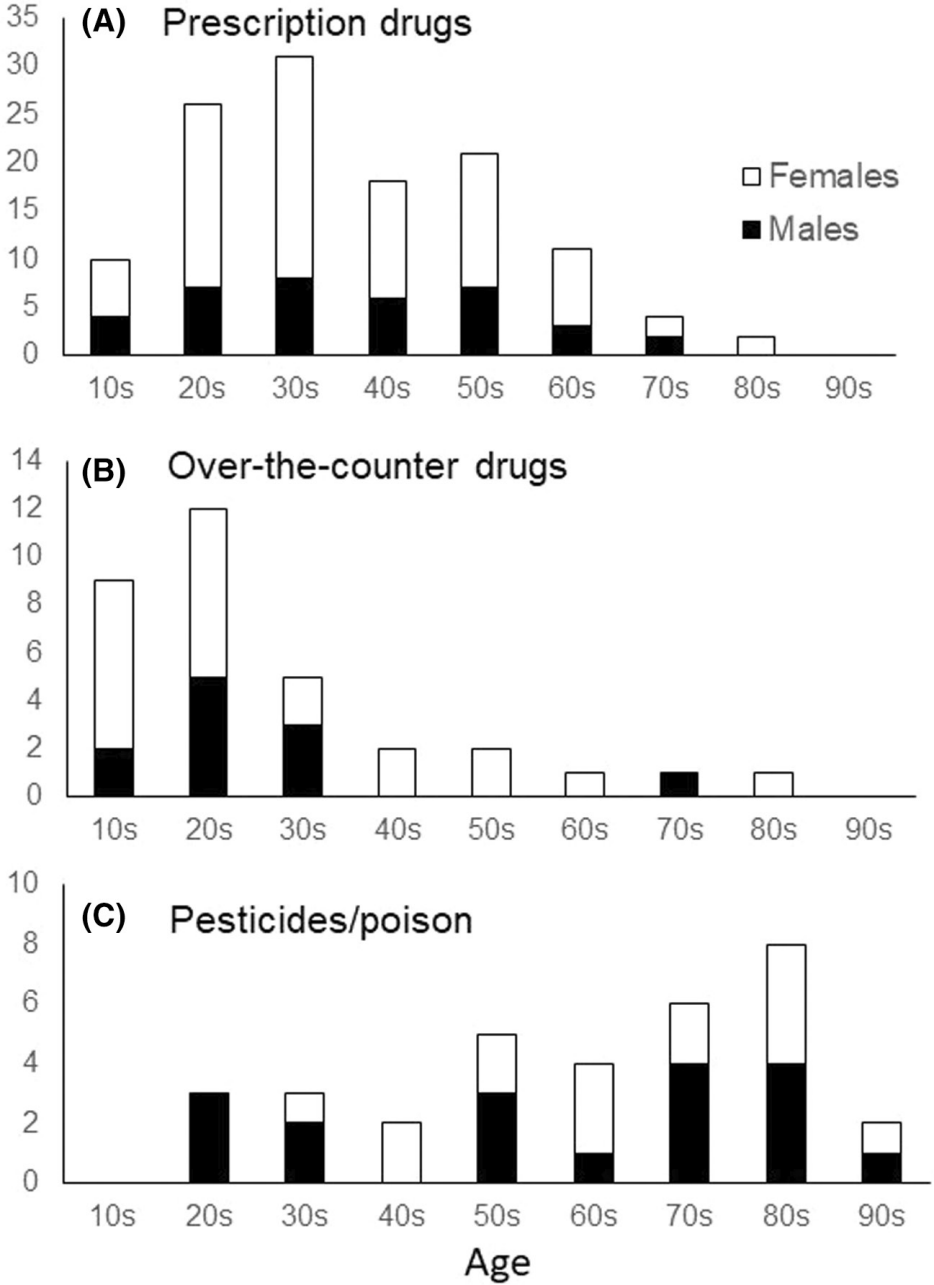
Histogram of the age distribution. Gray indicates females, and black indicates males. The top diagram (A) shows the age distribution of suicide attempts with prescription drugs, the middle diagram (B) shows the age distribution of suicide attempts with over‐the‐counter drugs, and the bottom diagram (C) shows the age distribution of suicide attempts with pesticides and poisons.

## 2 METHODS

In March 2020, a suicide reprogramming prevention team (DJ Project) was established in the psychiatry departments of two university hospitals in Tochigi Prefecture (Dokkyo Medical University Hospital and Jichi Medical University Hospital) to begin providing emergency psychiatric support for the prevention of suicide reattempts. Between April 2020 and March 2022, 250 patients with suicide attempts were transported to the two hospitals. There were 96 males (38.4%) and 154 females (61.6%). The mean age was 43.5 ± 20 years, and both males and females were most often in their 20s. The most common psychiatric diagnosis was mood disorders (F3) (34.1%). We determined what the patients had taken based on medicine shells provided by family members and from family members' testimonies. Data on sex, age, motive for suicide, means of suicide attempt, psychiatric diagnosis, length of hospital stay, and place of discharge were retrospectively analyzed. In addition, clinical characteristics were compared according to the three suicide attempt methods: prescription drugs, over‐the‐counter drugs, and pesticides/poisons. The ANOVA and chi‐square tests were used for statistical analysis, and *p* < 0.05 was considered significant. Multiple comparisons were performed using Tukey's test.

This study was approved by the Clinical Research Review Committees of Dokkyo Medical University Hospital, and consent was obtained from the subjects by opt‐out.

## 3 RESULTS

The most common means of suicide attempt were “prescription drugs” (43.9%), “over‐the‐counter drugs” (11.8%), “large doses of an unspecified medication” (1.8%), “pesticides/poison” (11.8%), “jumping” (9.6%), “stabbing/bladed instrument” (6.4%), “hanging” (6.4%), “carbon monoxide” (2.5%), “burning” (2.1%), “entering water” (1.4%), “alcohol” (1.4%), and “accident” (0.7%). The average age of the patients by means of suicide attempt was 40.5 years for “prescription drugs,” 30.2 years for “over‐the‐counter drugs,” 43.2 years for “OD with unknown content,” 63.5 years for “pesticide/poison,” 37.0 years for “jumping,” 50.1 years for “stabbing/bladed instrument,” 50.5 years for “hanging oneself,” 42.7 years for “carbon monoxide,” 59.5 years for “burning oneself,” 40.0 years for “water entry,” 42.5 years for “alcohol,” and 53.0 years for “accident.”

For each means of suicide attempt, there was a significant difference (*p* = 5.96 × 10^−14^) in age among patients who had taken “prescription drugs,” “over‐the‐counter drugs” and “pesticides/poisons” (Table 1 and Figure 1). Post hoc analyses revealed that the mean age of patients who had taken “prescription drugs” was significantly (*p* = 1.00 × 10^−4^) higher than that in patients who had taken “over‐the‐counter drugs,” and the mean age of patients who had taken “pesticides/poisons” was significantly (*p* = 5.10 × 10^−9^) higher than that of patients who had taken “prescription drugs.” In addition, the mean age of patients who had taken “over‐the‐counter drugs” was significantly (*p* = 5.35 × 10^−9^) higher than that of patients who had taken “pesticides/poisons.”

**TABLE 1:** Characteristics of patients among three means of suicide attempts.

	Prescription drugs	Over‐the‐counter drugs	Pesticides/poison	Significant
	*n* = 122	*n* = 23	*n* = 33	
Age (years)	40.2 (16.4)	26.0 (15.0)	63.5 (20.8)	*p* = 5.97 × 10^−14^
Females (*n*, %)	86 (70.5)	15 (65.2)	15 (45.4)	*p* = 0.028
Hospitalization (*d*)	9.8 (20.5)	14.8 (21.3)	16.5 (21.0)	*p* = 0.213
Motives for suicide attempts				
Mental illness (*n*, %)	13 (10.7)	0 (0.0)	4 (12.1)	*p* = 0.240
Financial problems (*n*, %)	14 (11.5)	2 (8.7)	4 (12.1)	*p* = 0.913
Interpersonal relations (*n*, %)	79 (64.8)	20 (87.0)	11 (33.3)	*p* = 1.27 × 10^−4^
Health problems (*n*, %)	5 (4.1)	2 (8.7)	7 (21.2)	*p* = 0.005
Work problems (*n*, %)	11 (9.0)	3 (13.0)	1 (3.0)	*p* = 0.380

Motives for suicide included mental illness, financial problems, life problems, health problems, and work problems. Significant frequency differences were found among these means of suicide attempts for interpersonal relationships (*p* = 1.27 × 10^−4^) and health problems (*p* = 0.005) as the motives for suicide (Table 1). The most common motive for suicide among both male and female patients was life problems such as family relationships. The average length of hospital stay was 16.1 ± 25 days. The most common discharge destination was home (56.8%).

## 4 DISCUSSION

In this study, for each means of suicide attempt by medication, there was a significant difference in the age of the patients who had taken “prescription drugs,” “over‐the‐counter drugs,” and “pesticides/poisons.” Among these, over‐the‐counter drugs were more common among young adults, while prescription drugs tended to be used by young adults and adolescents and pesticides tended to be used by older adults. This suggests that over‐the‐counter drugs are easily accessible in Japan and that doctors may prescribe them easily.

The percentage of ODs (including prescription, over‐the‐counter, and other ODs) among patients with suicide attempts in this study was 57.3%, an extremely high percentage compared to previous studies. A previous study found that in 2000, approximately one‐quarter of suicide attempts in the U.K.^5^ In addition, according to the Centers for Disease Control and Prevention (CDC), approximately 15% of suicide attempts in the United States in 2016 were caused by ODs, and the percentage of suicide attempts by using opioids increased from 2.2% in 1999 to 4.3% in 2014%.^6^

A higher percentage of females than males was also found to have used prescription and over‐the‐counter medication (69.9% and 66.7%, respectively), while males were slightly more likely to have used pesticides/poisonous substances (54.5%). This seems to be similar to previous reports in which men choose relatively more lethal methods of suicide attempts compared to women.^7^, ^8^ It has long been recognized that the characteristics of suicidality in children and adolescents differ from those in adults, and differences in the means of suicide attempts across age groups may provide useful criteria for the evaluation and treatment of unconscious patients with suicide attempts.

In addition, the tendency to use relatively lethal means of suicide, such as pesticides and burning, was more pronounced in the older age groups, consistent with the report by Hawton et al. reporting that suicide rates per population are generally higher in older age groups than in younger age groups and that the proportion of deaths (attempts) is also higher.^8^, ^9^, ^10^

This study had some limitations. There were several cases in which more than one means was used at the same time. In such cases, we counted them both and performed the statistics. In addition, the findings may not be applicable outside Japan because domestic circumstances may be involved, or the cultural backgrounds may be different.

In conclusion, the results of this study showed that overdose and pesticide/toxin use were characterized by age and sex. It is thought that pesticide use should be considered first thing, especially when patients aged 50 years and over are brought to the hospital with impaired consciousness due to suicide attempts.

## AUTHOR CONTRIBUTIONS

NYF and SS designed the study. MS, AN, and YN collected data. KS and SS supervised the project. TS and NYF wrote the manuscript. All authors reviewed and approved the final draft.

## FUNDING INFORMATION

Not applicable.

## CONFLICT OF INTEREST STATEMENT

The authors declare no conflict of interest.

## ETHICS STATEMENT

Approval of the research protocol by an Institutional Reviewer Board: The study protocol was approved by the institutional ethics committees.

Informed consent: This study was posted on the institution website as a clinical study using opt‐out consent.

Registry and the Registration No. of the study/Trial: N/A.

Animal studies: N/A.

## Data Availability

The Ethics Committee of the Dokkyo Medical University Hospital has set restrictions on data sharing because the data contain potentially identifying or sensitive patient information. Please contact the institutional review board of the ethics committee of the Dokkyo Medical University Hospital for data requests. Upon request, the ethics committee will decide whether to share the data.
